# Another Veterinary Journal—Another Veterinary College

**Published:** 1899-09

**Authors:** 


### EDITORIAL.

### ANOTHER VETERINARY JOURNAL—ANOTHER VETERINARY COLLEGE.

With the closing days of the summer season comes the first issue of a new journalistic venture on the Pacific coast and a veterinary college opening in the Southern field.

New York and Philadelphia are too far distant to cover the field of the Pacific slope's needs of the profession in the field of journalism, and to this crying need there comes the response of the new journal. It is edited and launched by a member of the profession who has had so varied a career as to give promise of a peculiar fitness for the work. In fact, if experience in journalistic lawsuits, Bureau of Animal Industry controversies, State examining board selections and appointments, association circle difficulties, and veterinary college direction, as well as in all the aspects of the daily difficulties in routine practice—surely an array of experiences and qualifications that may be made a powerful influence in veterinary journalism. The first issue, modest in its promises, with ample room to enlarge and grow to fill the profession's needs, and at a reduced price from that maintained by the older journals, it makes one grave determination that is to be most highly commended, in that it does not propose to wreck itself on the shoals that proved the destruction of many former undertakings—of giving more for the money than it received.

We welcome this new periodical to the field; there is room at the top, and we sincerely trust that the experiences of all prior journalistic progenitors, of having to carry it along for many years out of their own pockets, may not befall our fellow-editor.

The visit of the National Association to Nashville, to awaken the needs of the South in veterinary work, has borne fruit long before it was thought the seed sown there could germinate; but this is a rushing, progressive country, and the needs of the South are pressing and importunate. The long period of the other schools, with their three-year curriculums and large corps of instructors, would not meet the exacting demands created by our visit in 1897; hence this school at Nashville starts out on a two-years' basis and with a non-graduate as its promoter and head. In the initial announcement the chief director did not consider it essential to secure the consent or acquiescence of those announced as the Faculty; hence the prompt repudiation of two reputable veterinarians of having any connection with the same, and the fact of other names announced not being known in the veterinary or medical teaching world. This school, broader than its predecessors, will have a farrier's department, and diplomas for shoers will also be given, and a large shoeing-forge connected with the school will add to its income and afford the citizens of Nashville a special opportunity of having their animals shod.

The fees are attractive, and the school insures many other advantages, attractively spread forth in the catalogue, that are sure to be appreciated by a long-suffering section of our country, and which now for the first time is afforded a get-there-quick supply of veterinarians.

